# Flex Sensor Compensator via Hammerstein–Wiener Modeling Approach for Improved Dynamic Goniometry and Constrained Control of a Bionic Hand

**DOI:** 10.3390/s19183896

**Published:** 2019-09-10

**Authors:** Syed Afdar Ali Syed Mubarak Ali, Nur Syazreen Ahmad, Patrick Goh

**Affiliations:** School of Electrical and Electronic Engineering, Engineering Campus, Universiti Sains Malaysia, 14300 Nibong Tebal, Pulau Pinang, Malaysia

**Keywords:** flex sensor, hand gesture, Hammerstein–Wiener

## Abstract

In this paper, a new control-centric approach is introduced to model the characteristics of flex sensors on a goniometric glove, which is designed to capture the user hand gesture that can be used to wirelessly control a bionic hand. The main technique employs the inverse dynamic model strategy along with a black-box identification for the compensator design, which is aimed to provide an approximate linear mapping between the raw sensor output and the dynamic finger goniometry. To smoothly recover the goniometry on the bionic hand’s side during the wireless transmission, the compensator is restructured into a Hammerstein–Wiener model, which consists of a linear dynamic system and two static nonlinearities. A series of real-time experiments involving several hand gestures have been conducted to analyze the performance of the proposed method. The associated temporal and spatial gesture data from both the glove and the bionic hand are recorded, and the performance is evaluated in terms of the integral of absolute error between the glove’s and the bionic hand’s dynamic goniometry. The proposed method is also compared with the raw sensor data, which has been preliminarily calibrated with the finger goniometry, and the Wiener model, which is based on the initial inverse dynamic design strategy. Experimental results with several trials for each gesture show that a great improvement is obtained via the Hammerstein–Wiener compensator approach where the resulting average errors are significantly smaller than the other two methods. This concludes that the proposed strategy can remarkably improve the dynamic goniometry of the glove, and thus provides a smooth human–robot collaboration with the bionic hand.

## 1. Introduction

Hand gesture recognition refers to the process of understanding the mathematical interpretation of the hand’s movement by a computing device [1]. It is one of the popular research topics in the past few decades due to the rapid advancements in sensor and smart device technologies [2,3,4]. Its applications are not just limited to human–computer or human–machine interaction [5], but also include a diverse range of fields such as sign language recognition [6], clinical rehabilitations [7,8], human–robot collaborations [9,10], gaming and virtual reality control [11]. In a typical hand gesture recognition system, the initial stage is the data acquisition which can be performed either via vision-based [12] or non-vision-based [13] techniques. Both have their own advantages and disadvantages regarding their applications. A hybrid approach which combines the two methods has also been employed in some specific areas that require high speed and precision such as augmented reality technologies [14].

The vision-based technique, as the name suggests, uses cameras or optical sensors to capture hand gestures. A notable superiority of this technique is that it eliminates the need for using wearable interfaces or multi-sensory devices, hence providing a natural interaction with the computer. In other words, the minimal interference offered allows the user to perform any hand motion in the most convenient way possible. Nonetheless, the tracking performance is greatly affected by environmental factors such as illumination or lighting variations, cluttered background, and interruption from other moving objects in the scene particularly with the same hand skin color. Other major problems caused by the user’s motion include out-of-range detection, high-speed movement, and self-occlusion. Such circumstances entail high specification cameras [15,16] or optical sensors such as Leap Motion controller [17,18,19,20,21,22] and Microsoft Kinect sensors [23] to enhance its performance and robustness. In some cases, multiple cameras at different view angles and positions are required, which may eventually increase the computational burden and associated costs [24,25]. This can also lead to bulkiness and may not be desirable for mobile applications.

The non-vision-based method, on the other hand, has the advantage of not being susceptible to occlusion or environmental factors. This approach is also frequently termed as sensor-based method, although literally it encompasses any kind of sensing devices but image or vision sensors. The most commonly used interface is the glove-based gestural system which is constructed by several sensors attached to a cloth glove, a computing device for data processing and transmission, and a power supply. A major benefit of this method over the vision-based technique is its fast response and precise tracking [10]. The performance can be boosted by increasing the number of sensing devices, but it will pose a trade off with the size, power consumption, building costs, and most importantly, user convenience. In the non-vision-based technique, different sensors serve to capture different types of hand gestures, such as palm orientation, wrist movement, hand rotation, and fingers goniometry. The first three types mentioned can be simply registered using accelerometers, gyroscopes or inertial measurement units (IMUs). With proper calibrations, the combination of the gestures allows the overall 3D motion to be captured with a good performance.

The predominant gesture recognition that is of interest in much research and yet remains a significant technical challenge is nevertheless the finger goniometry [6,8,26,27]. Goniometry in general refers to the measurement of angles created at human joints by the bones of the body [28]. A crucial stage before the goniometry can be acquired is the procedure to model the hand, where the variations can be recorded either temporally, spatially, or both, depending on the target applications. Via non-image-based data acquisition approach, the 2D hand modeling for temporal or spatial pattern assessment can be accomplished by using the flex sensor as it has a prominent advantage of being able to change its resistance when bent. This characteristic makes it suitable to be positioned on the finger’s joints where the goniometry can be correlated with the sensor’s bend angle. Plus, this type of sensor only requires a simple electronic interface to translate the resistance into a voltage output. For specific applications that only need a nonlinear gesture mapping to discrete interpretations [29], for instance, the sign language recognition [24,30], the difficulty level of the task will be minimized as a static analysis will suffice. Simply put, only the initial and final postures of the fingers can be prioritized for further extraction and classifications. For some others where a linear gesture mapping is the main design requirement, monitoring a patient’s hand’s functionality or motor performance [31,32], as an example, the task will be relatively more cumbersome. In this scenario, if one is to use the flex sensor as the primary tool, a dynamic analysis with a high degree of accuracy within the sensor data acquisition subsystem is needed to provide a precise mapping to the target applications.

One major challenge in using the flex sensor for dynamic goniometry purposes is correlating the flex sensor’s bending angle with the goniometry as the resistance tends to be time-varying and is prone to uncertainties particularly when sewn or attached to a cloth glove [33,34]. Moreover, some cloth materials used may provide stiffness or are bound to wear and tear, which consequently cause erroneous representation of the bending angle [32]. Owing to this, several data processing algorithms have been introduced in the literature that operate on the sensor raw output to improve the gesture tracking performance. One of the most common approaches is by using machine learning tools such as artificial neural network [35,36] and Hidden Markov Model [37,38]. While this can provide flexibility of training and verification, and is useful to detect nonlinear relationships between variables, a large training data set is required and many free parameters need to be optimized in order to obtain a model with great accuracy [39]. In some cases where a high sample rate in data acquisition is used, or the sensor output is frequently perturbed by the Gaussian noise, Kalman filtering approach will be better suited, provided that the sensor bending angle has been well calibrated with the static goniometry [26]. Another recent technique via first principle modeling is proposed in [27] where the mathematical representation that relates the flex sensor’s resistance and the bending angle is derived based on the understanding of the system’s underlying physics. The advantage of this approach is the detailed insight into the behavior of the system and hence leads to a better prediction on the performance, whereas the disadvantage is the difficulties in determining the phenomenological parameters caused by internal and external disturbances.

It is also worth to note that even though similar types of sensors are used in a glove-based gestural system, a straightforward comparison between the methods that have been introduced over the years may not be appropriate due to different application-specific tasks and design requirements particularly those concerning the types and speed of gestures, sampling time, sensor locations, as well as data mapping [13]. In this work, a new control-centric approach is introduced to model the characteristic of flex sensors on a goniometric glove, which is designed to capture the user hand gesture that can be used to wirelessly control a bionic hand. The main technique employs the inverse dynamic model strategy along with a black-box identification for the compensator design, which is aimed to provide an approximate linear mapping between the raw sensor output and the dynamic finger goniometry. To smoothly recover the goniometry on the bionic hand’s side during the wireless transmission, the linearity of the mapping needs to be improved. Hence, we propose a Hammerstein–Wiener model to enhance the structure of the compensator, which consists of a linear dynamic system and two static nonlinearities. The linear system is constructed by simplifying the dynamic model from the inverse dynamic design technique, while the static nonlinearities are introduced based on the constraints of the bionic hand, and to account for the uncertain behavior of the sensors as well as the unmodeled dynamics from the black-box identification method. A series of real-time experiments involving several hand gestures have been conducted to analyze the performance of the proposed method. In the experiments, the goniometric speed for each finger is controlled at approximately $83^{\circ }$/s for all gestures. The associated temporal and spatial data from both the glove and the bionic hand are recorded via an image processing technique, and the performance is evaluated in terms of the integral of absolute error between the glove’s and the bionic hand’s dynamic goniometry. The proposed method is also compared with the raw sensor data, which has been preliminarily calibrated with the finger goniometry, and the Wiener model, which is based on the initial inverse dynamic design strategy. Experimental results with several trials for each gesture show that the raw sensor data result in average errors between 515${}^{\circ }$s and 1347${}^{\circ }$s, whereas for the Wiener model, the average errors lie between 186${}^{\circ }$s and 370${}^{\circ }$s, which are well below the range from the raw data. A significant improvement is obtained via the Hammerstein–Wiener compensator where the resulting average errors are no greater than $102^{\circ }$s. This concludes that the proposed strategy can remarkably improve the dynamic goniometry of the glove, and thus, provides a smooth human–robot collaboration with the bionic hand.

The remainder of the paper proceeds as follows: Section 2 discusses the background and statement of the problem concerning the nonlinear characteristics of the flex sensor from a preliminary analysis. The bionic hand description, the goniometric glove structure, the proposed compensator design method, and the experimental setup are explained in detail in Section 3. Section 4 presents the experimental results from several hand gesture tests, and the average error for each method. The results are finally concluded and discussed in Section 5, together with future work.

## 2. Background and Problem Statement

**Figure 1:**
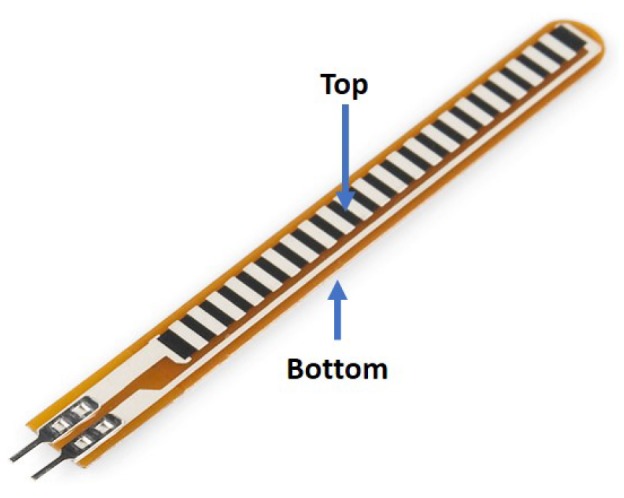
A 2.2${}^{\prime \prime }$ unidirectional flex sensor.

**Figure 2:**
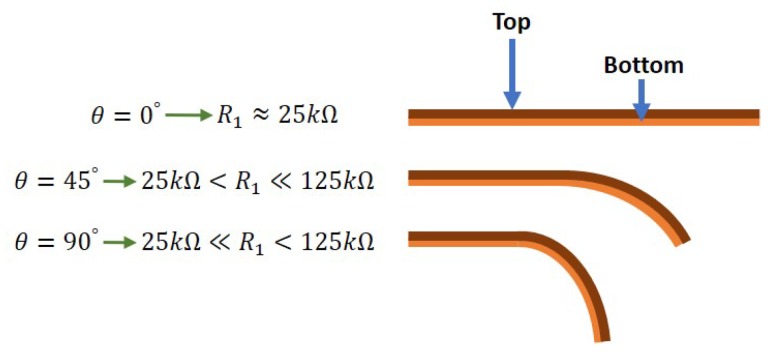
Illustration on the relation between the flex sensor bending angle, θ, and the resistance, $R_{1}$.

A flex sensor is basically a variable resistor that reacts to bends, i.e., it changes its resistance when the bending angle increases. The flex sensor considered in this work is of unidirectional type as shown in Figure 1. When in default position (i.e., flat/relaxed), the resistance measures around 25 kΩ, and may increase up to 125 kΩ as it bends towards $180^{\circ }$. This is illustrated as in Figure 2 where θ and $R_{1}$ denote the bending angle and resistance, respectively.

The sensor can be configured to act as a voltage divider where the corresponding output, $V_{out}$ is simply:

(1) $$V_{out}=\frac{R_{1}}{R_{1}+R_{2}}V_{in}.$$

Theoretically, the value of $R_{1}$ (in kΩ) relates to the bending angle as follows:

(2) $$\begin{array}{c} R_{1}=100\cdot \frac{\theta }{180}+25. \end{array}$$

**Figure 3:**
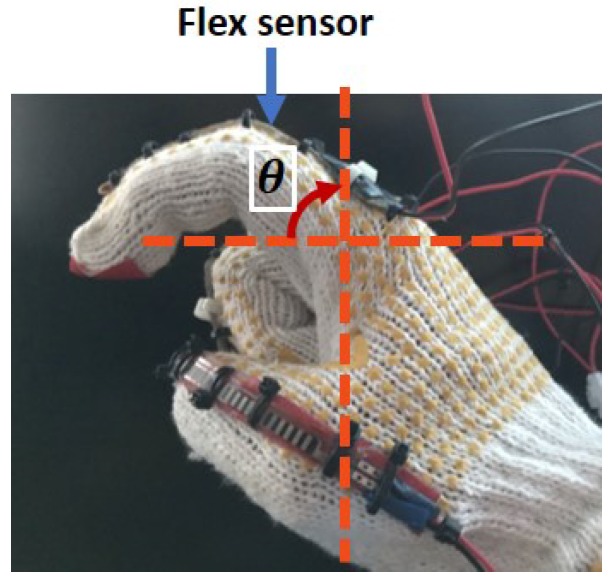
A preliminary analysis to investigate the correlation between the sensor output voltage and the bending angle when the sensor is tied on a cloth glove.

**Figure 4:**
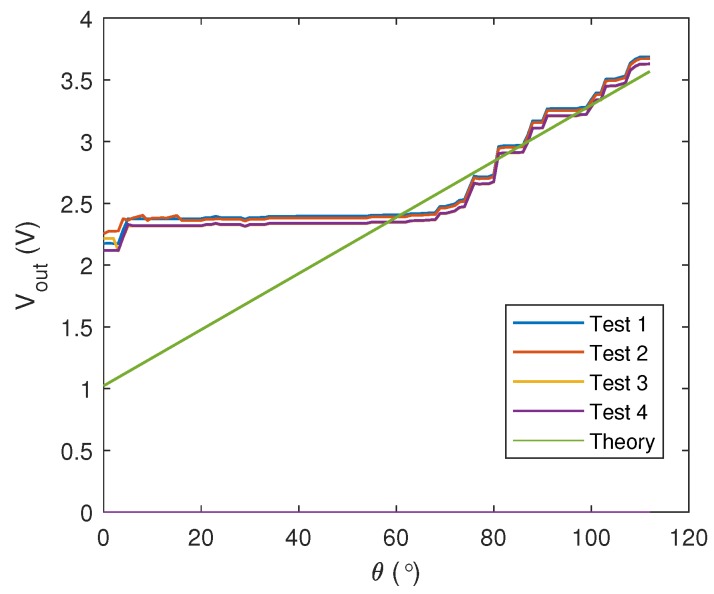
Comparisons between real and theoretical sensor output voltages with respect to the bending angle.

When the value of $R_{2}$ is fixed, we have the relation

(3) $$\begin{array}{c} V_{out}=\frac{100\theta +4500}{100\theta +180(25+R_{2})}V_{in}, \end{array}$$

which implies a linear relationship between θ and $V_{out}$. Nevertheless, when the sensor is attached to a moving finger, the exact value of θ will not be smoothly recovered due to the non-smooth finger’s movement. Also, the position of the sensor with respect to the finger may additionally affect the resistance, leading to unpredictable behavior. A preliminary analysis has been conducted to investigate the correlation between the sensor output voltage and the bending angle when the sensor is tied on a cloth glove as shown in Figure 3. Results from four tests when $R_{2}$ = 35 kΩ and $V_{in}$ = 5 V have been recorded in Figure 4, which are also compared with the theoretical values from Equation (3). From the figure, the inconsistencies of the sensor output and the mismatch with the theoretical values reflect the existence of nonlinearities and uncertainties in the sensor model itself.

The focus of this research is to propose a compensator for the goniometric glove with the aforementioned flex sensors which can dynamically recover the gesture of each finger. To wirelessly control a bionic hand using the recovered gesture, the glove has also been preliminarily designed by taking into account the constraints of the bionic hand. The main strategy to achieve this objective is explained in detail in the next section.

## 3. Methodology and Experimental Setup

### 3.1. Bionic Hand Description

**Figure 5:**
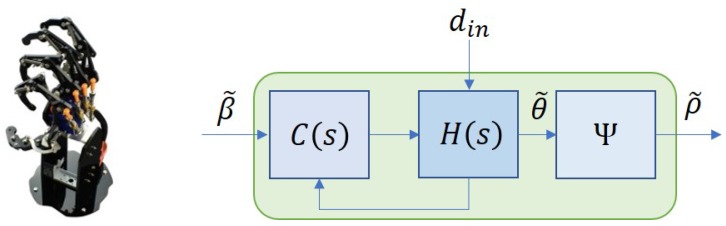
Bionic hand (**left**); Closed-loop control structure of the bionic hand (**right**).

Throughout this paper, the index i=1,2,3,4 and 5 will represent signals associated with the thumb, pointer, middle, ring, and pinky fingers, respectively. The bionic hand system used in this work is controlled by five dc motors where the input, $\tilde{\beta }={\left[\beta_{1},\beta_{2},\beta_{3},\beta_{4},\beta_{5}\right]}^{T}$, is supplied by the signals from an ATMega microcontroller (denoted as μC1). The overall closed-loop system can be illustrated as in Figure 5 where C(s) and H(s) denote the proportional-integral-based motor controller and the bionic hand, respectively. Each motor is assigned to control the flexion or extension of each finger from the metacarpophalangeal (MCP) joint, and the system is subject to possible bounded disturbances, $d_{in}$. In this work, the effects of the disturbance are assumed only in terms of slow and slightly nonlinear movements due to deadzones or frictions, and do not lead to instability of the system.

**Figure 6:**
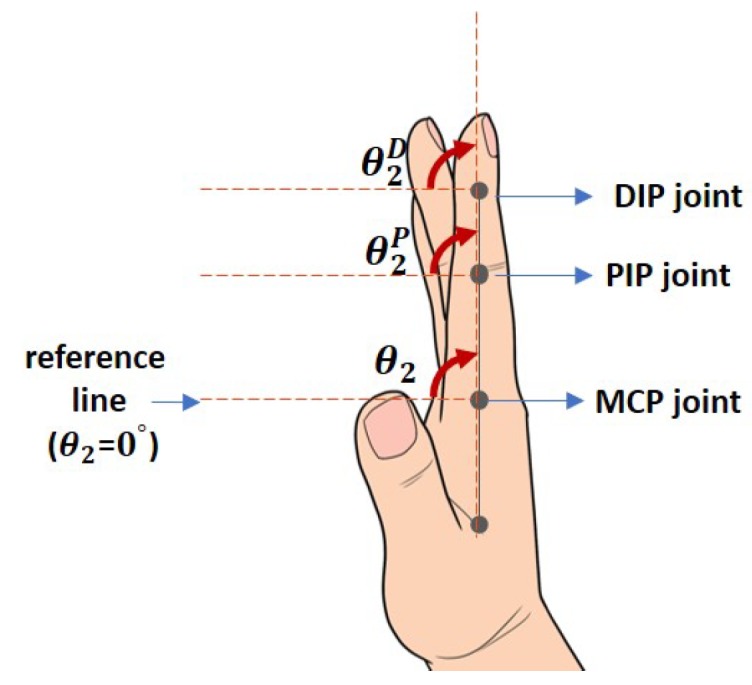
Illustration on the goniometry of the pointer finger and its’ reference line. The figure shows $\theta_{2}=90^{\circ }$.

**Figure 7:**
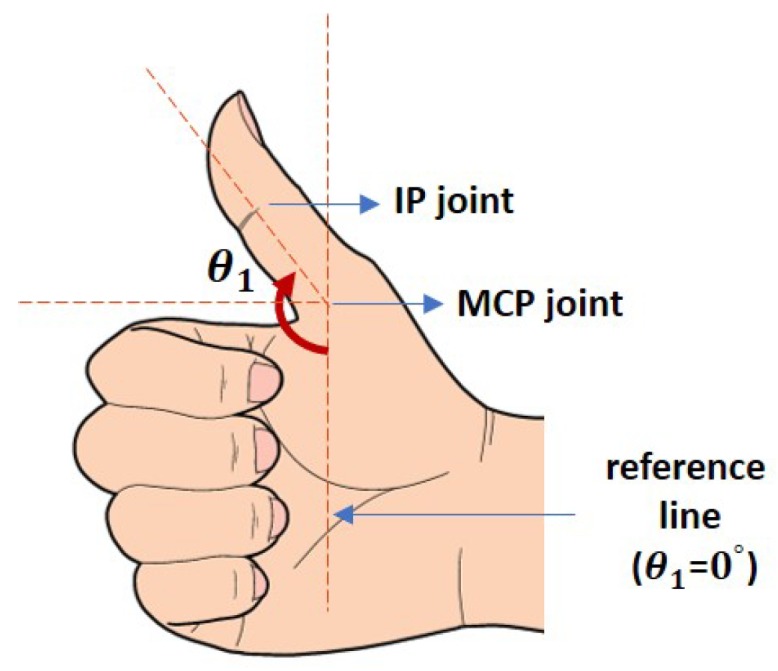
Illustration on the goniometry of the thumb and its’ reference line. The figure shows $\theta_{1}=135^{\circ }$.

The bionic hand has also been designed to mimic the normal behavior of finger movements which can be mathematically described by

(4) $$\begin{array}{cc} \theta_{i}^{D} & \approx \frac{2}{3}\theta_{i}\;\mathrm{for}\;i=2,3,4,5; \end{array}$$

(5) $$\begin{array}{cc} \theta_{i}^{P}\approx \frac{3}{4}\theta_{i}\;\mathrm{for}\;i & =2,3,4,5\;\mathrm{and}\;\theta_{i}^{P}\approx \frac{1}{2}\theta_{i}\;\mathrm{for}\;i=1 \end{array}$$

where $\theta_{i}^{D}$, $\theta_{i}^{P}$ and $\theta_{i}$ are the goniometry measured at distal interphalangeal (DIP), proximal interphalangeal (PIP) and MCP joints respectively (see the corresponding counterparts in Figure 6). Equations (4) and () imply that the fingers’ bending angles from DIP and PIP joints are often influenced by the movement from the MCP joints [26]. For i=2,3,4 and 5, the goniometry share the same reference line as illustrated by the pointer finger in Figure 6, while for i=1, the reference line is $-90^{\circ }$ below that of those for i=2,3,4,5 (shown in Figure 7), and only MCP and interphalangeal (IP) joints exist.

The overall movement is controlled by the motors attached at the MCP joints, which automatically changes $\theta_{i}^{D}$ and $\theta_{i}^{P}$ when $\theta_{i}$ is changed. Ideally, $\tilde{\theta }={\left[\theta_{1},\theta_{2},\theta_{3},\theta_{4},\theta_{5}\right]}^{T}$ should follow the reference command, $\tilde{\beta }$, but it may not always be the case due to the presence of $d_{in}$ which can randomly enter the system at any time instance. Apart from that, a constraint, $\Psi =diag(\psi_{1},\psi_{2},\psi_{3},\psi_{4},\psi_{5})$, is imposed on $\tilde{\theta }$ to resemble the typical range of joint motions, which is described as

(6) $$\begin{array}{cc} \rho_{i}=\Psi_{i}\left(\theta_{i}\right) & =\left\{\begin{array}{cc} \theta_{L} & \;\mathrm{if}\;\theta_{i}<\theta_{L}\\ \theta_{i} & \;\mathrm{if}\;\theta_{L}\leq \theta_{i}\leq \theta_{U}\\ \theta_{U} & \;\mathrm{if}\;\theta_{i}>\theta_{U} \end{array}\right. \end{array}$$

where $\theta_{L}$ and $\theta_{U}$ denote the lower and upper bounds, respectively. For i=1 (i.e., thumb), the movement is limited by $\theta_{L}=90^{\circ }$ and $\theta_{U}=170^{\circ }$, whereas for i=2,3,4,5, the bounds are $\theta_{L}=28^{\circ }$ and $\theta_{U}=113^{\circ }$.

### 3.2. Goniometric Glove with Compensators

**Figure 8:**
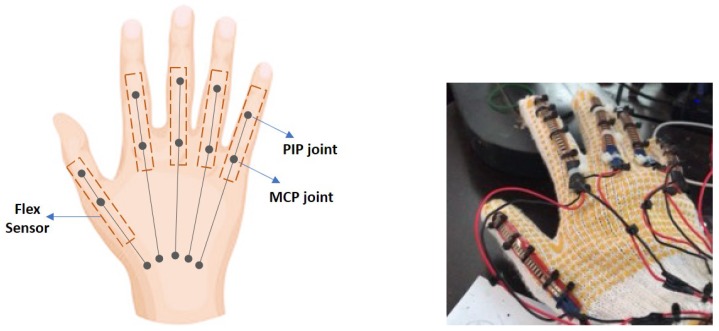
Positions of flex sensors with respect to the MCP and PIP joints (**left figure**); Flex sensors attached to the goniometric glove (**right figure**).

**Figure 9:**
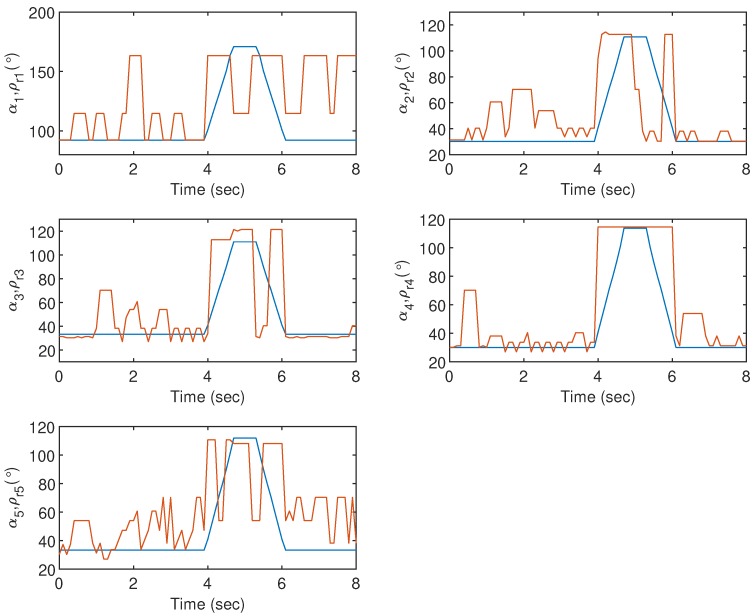
Goniometric glove response with respect to a grab-release-grab movement. The finger goniometry and the raw sensor outputs are represented by $\rho_{ri}$ (blue line) and $\alpha_{i}$ (orange line) respectively.

In this research, the goniometric glove is made of cotton with a thickness of around 2 mm. Preliminary analyses with a grab and release movement have been conducted to investigate suitable positions of the sensors on the goniometric glove. Let ${\tilde{\rho }}_{r}={\left[\rho_{r1},\rho_{r2},\rho_{r3},\rho_{r4},\rho_{r5}\right]}^{T}$ be the angles measured at the MCP joints of the goniometric glove. An image processing technique in MATLAB is used to capture ${\tilde{\rho }}_{r}$. Figure 8 (left) shows suitable positions of the sensors with respect to the MCP and PIP joints which can register an acceptable and predictable goniometry for each finger. The outputs of the sensors are connected to analog pins of an ATMega microcontroller (denoted as μC2) with a sample rate of 10 Hz. We denote the raw sensor values read by μC2 as $\tilde{\alpha }={\left[\alpha_{1},\alpha_{2},\alpha_{3},\alpha_{4},\alpha_{5}\right]}^{T}$, where each $\alpha_{i}$ ranges from 0 to 1023. The goniometric glove response in one of the tests is shown in Figure 9.

From Figure 9, it can be observed that when the sensors are positioned as depicted in Figure 8, the response does not deviate far from the fingers’ goniometry. On the other hand, it also suggests that the bionic hand requires a good precompensator to minimize the error between the goniometry and the sensors’ response.

To this end, we propose a dynamic compensator, P(s), which is constructed based on the inverse dynamic approach where the structure is designed using the inverse of the internal model that characterizes the behavior of ${\tilde{\rho }}_{r}$ and $\tilde{\alpha }$. The black-box system identification technique is used to estimate the dynamics of the model where the input is fed from the value of $\tilde{\alpha }$ while the output is the value from ${\tilde{\rho }}_{r}$. The highest accuracy from several datasets from the black-box system identification is approximately 70%, and the model with the highest accuracy is given by a linear time-invariant (LTI) model, $P∼(A_{p},B_{p},C_{p},D_{p})$ where $A_{p}=$diag$\left(A_{1},A_{2},A_{3},A_{4},A_{5}\right)$, $B_{p}=$diag$\left(B_{1},B_{2},B_{3},B_{4},B_{5}\right)$, $C_{p}=$diag$\left(C_{1},C_{2},C_{3},C_{4},C_{5}\right)$, with

$$\begin{array}{cc} A_{1} & =\left[\begin{array}{ccc} -84.51 & -56.92 & -21.02\\ 32 & 0 & 0\\ 0 & 16 & 0 \end{array}\right],\;A_{i}=\left[\begin{array}{cc} -31.6 & -14.7\\ 16 & 0 \end{array}\right],\;\mathrm{for}\;i=2,3,4,\\ A_{5} & =\left[\begin{array}{ccc} -7863 & -673.9 & -81.33\\ 512 & 0 & 0\\ 0 & 64 & 0 \end{array}\right],B_{1}=\left[\begin{array}{c} 4\\ 0\\ 0 \end{array}\right],\;B_{i}=\left[\begin{array}{c} 4\\ 0 \end{array}\right],\;\mathrm{for}\;i=2,3,4,\;B_{5}=\left[\begin{array}{c} 8\\ 0\\ 0 \end{array}\right],\\ C_{1} & =\left[\begin{array}{ccc} 0 & 0 & 5.073 \end{array}\right],\;C_{2}=\left[\begin{array}{cc} 0 & 3.252 \end{array}\right],\;C_{3}=\left[\begin{array}{cc} 0 & 4.666 \end{array}\right],\;C_{4}=\left[\begin{array}{cc} 0 & 4.424 \end{array}\right],\;C_{5}=\left[\begin{array}{ccc} 0 & 0 & 11.27 \end{array}\right], \end{array}$$

and $D_{p}=$diag (0,0,0,0,0).

**Figure 10:**
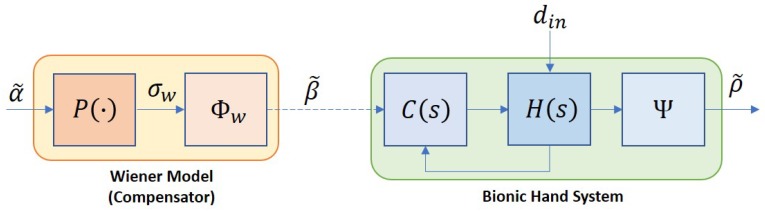
Bionic hand with a Wiener compensator.

As the output of the bionic hand system is constrained, a static nonlinearity, $\Phi_{w}={\left[\phi_{w1},\;\phi_{w2},\;\phi_{w3},\;\phi_{w4},\;\phi_{w5}\right]}^{T}$ block is included in the compensator to ensure the input to the bionic hand system stays within the range. The nonlinearity is described as

(7) $$\begin{array}{cc} \phi_{wi}\left(\sigma_{wi}\right) & =\left\{\begin{array}{cc} \sigma_{wl} & \;\mathrm{if}\;\sigma_{wi}<\sigma_{wl}\\ \sigma_{wi} & \;\mathrm{if}\;\sigma_{wl}\leq \sigma_{wi}\leq \sigma_{wu}\\ \sigma_{wu} & \;\mathrm{if}\;\sigma_{wi}>\sigma_{wu} \end{array}\right. \end{array}$$

where $\sigma_{wl}=90^{\circ }$ and $\sigma_{wu}=170^{\circ }$ when i=1, and $\sigma_{wl}=29^{\circ }$ and $\sigma_{wu}=112^{\circ }$ when i=2,3,4,5. The combination of P(s) and $\Phi_{w}$ in series forms a Wiener-type compensator, which is illustrated as in Figure 10.

**Figure 11:**
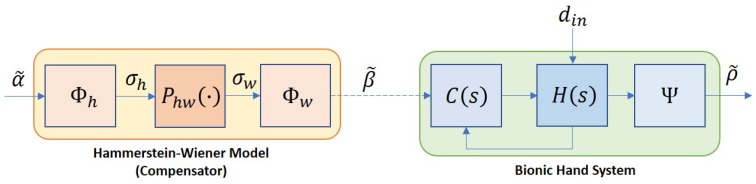
Overall control system structure with the Hammerstein–Wiener compensator.

The accuracy of 70% from the black-box system identification actually reflects the effects of nonlinearities in the model. To further enhance the tracking performance of the compensator, these effects need to be suppressed. Based on the variations of resistance in the preliminary analysis as shown in Figure 4, we propose a slight modification on the compensator where *P* is partitioned into two blocks as depicted in Figure 11, consisting of a simplified LTI model, $P_{hw}$, and another static nonlinearity, $\Phi_{h}={\left[\phi_{h1},\;\phi_{h2},\;\phi_{h3},\;\phi_{h4},\;\phi_{h5}\right]}^{T}$. The $P_{hw}$ is constructed based on the estimated dominant pole from *P*, which results in only first order linear model for each finger. The new configuration of the compensator is well-known as the Hammerstein–Wiener structure which, in general, is an LTI system in series with two static nonlinearities.

The simplified dynamic block of the compensator, $P_{hw}∼\left(A_{ph},B_{ph},C_{ph},D_{ph}\right)$, is constructed as follows:

$$\begin{array}{cc} A_{ph} & =\left[\begin{array}{ccccc} 54 & 0 & 0 & 0 & 0\\ 0 & 19 & 0 & 0 & 0\\ 0 & 0 & 19 & 0 & 0\\ 0 & 0 & 0 & 21 & 0\\ 0 & 0 & 0 & 0 & 15 \end{array}\right],\;B_{ph}=\left[\begin{array}{ccccc} 69 & 0 & 0 & 0 & 0\\ 0 & 24 & 0 & 0 & 0\\ 0 & 0 & 27 & 0 & 0\\ 0 & 0 & 0 & 28 & 0\\ 0 & 0 & 0 & 0 & 21 \end{array}\right],\;C_{ph}=\left[\begin{array}{ccccc} 1 & 0 & 0 & 0 & 0\\ 0 & 1 & 0 & 0 & 0\\ 0 & 0 & 1 & 0 & 0\\ 0 & 0 & 0 & 1 & 0\\ 0 & 0 & 0 & 0 & 1 \end{array}\right] \end{array}$$

and $D_{ph}=$diag (0,0,0,0,0), whereas for the static nonlinearity, it can be described as

(8) $$\begin{array}{c} \phi_{hi}\left(\alpha_{i}\right)=\left\{\begin{array}{cc} \alpha_{i}+\varepsilon_{l} & \;\mathrm{for}\;\alpha_{i}<-\varepsilon_{l}\\ 0 & \;\mathrm{for}\;-\varepsilon_{l}\leq \alpha_{i}\leq \varepsilon_{u}\\ \alpha_{i}-\varepsilon_{u} & \;\mathrm{for}\;\alpha_{i}>\varepsilon_{u}. \end{array}\right. \end{array}$$

where $\varepsilon_{l}=10$ and $\varepsilon_{u}=20$ when i=1,2,3,4, and $\varepsilon_{l}=10$ and $\varepsilon_{u}=30$ when i=5.

### 3.3. Experimental Setup

For wireless communication between the goniometric glove and the bionic hand, an HC-12 serial communication module is connected to μC1 as a receiver, and another similar module is connected to μC2 and configured as a transmitter. Six different sets of gestures are considered for the experiments as follows:

- Gesture 1: Grab-release-grab
- Gesture 2: Number two sign
- Gesture 3: Call sign
- Gesture 4: Okay sign
- Gesture 5: Mixed Gestures A
- Gesture 6: Mixed Gestures B

**Figure 12:**
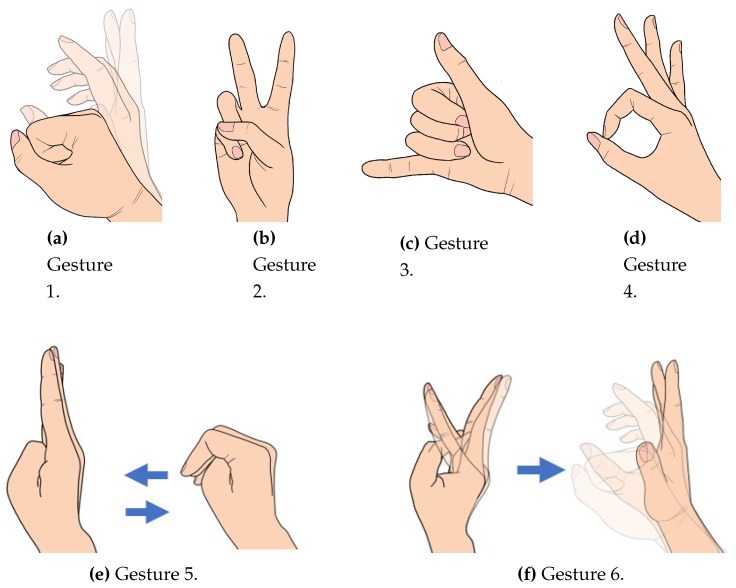
Hand gestures considered for the experiments.

**Figure 13:**
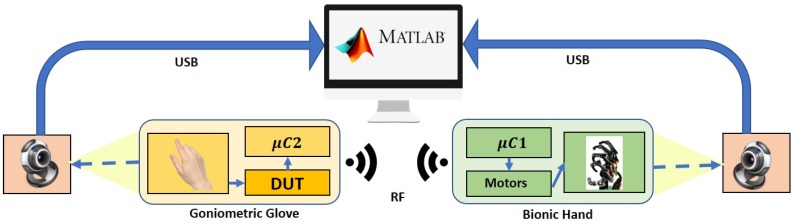
Schematic diagram for the experimental setup. The goniometric glove with the flex sensors is represented by “Device Under Test (DUT)”, and cameras are used together with image processing in MATLAB for performance evaluations.

These are illustrated as in Figure 12. The first four gestures involve at most three hand movement transitions, while the last two gestures involve six movement transitions. The experimental setup is depicted in Figure 13 where the performance of the overall system is evaluated based on the temporal mismatch between the glove’s and the bionic hand’s goniometry which are registered through cameras connected to a PC desktop via USB cables. The values of ${\tilde{\rho }}_{r}$ and $\tilde{\rho }$ are extracted via image processing techniques in MATLAB.

## 4. Experimental Results and Performance Evaluations

In the experiments, the bionic hand response, i.e., $\tilde{\rho }$, is compared when there is no compensator at all, and when Wiener and Hammerstein–Wiener compensators are implemented, which are denoted by “Raw”, “W”, and “HW” respectively in all figures. The response is also compared with the reference, ${\tilde{\rho }}_{r}$, which is from the glove goniometry. The experiments conducted focus on temporal analysis, and the goniometric speed for each finger is controlled at approximately $83^{\circ }$/s. A series of postures from the bionic hand are also recorded for a simple spatial analysis.

**Figure 14:**
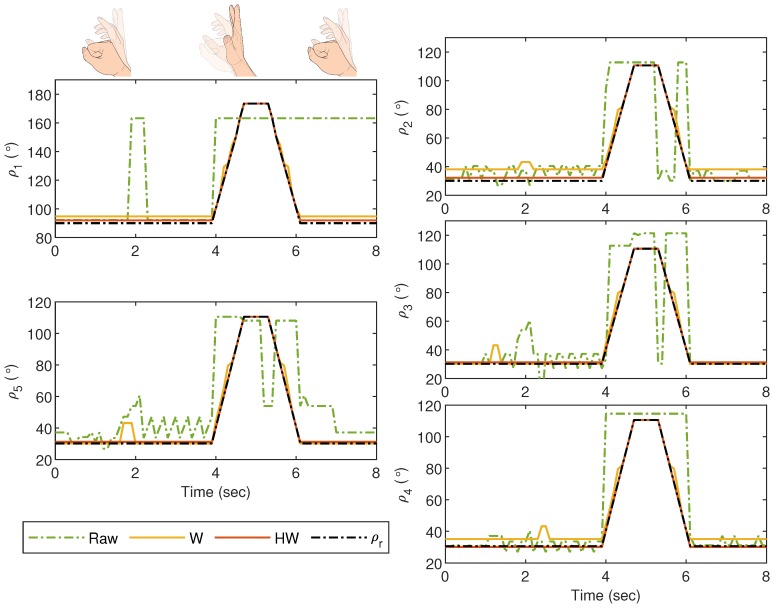
Gesture 1: The mismatch between the glove’s and bionic hand’s goniometry is significantly reduced by using Compensators W and HW. Compensator HW is seen to provide a better response as compared to Compensator W, particularly before t=4 s and after t=6 s.

**Figure 15:**
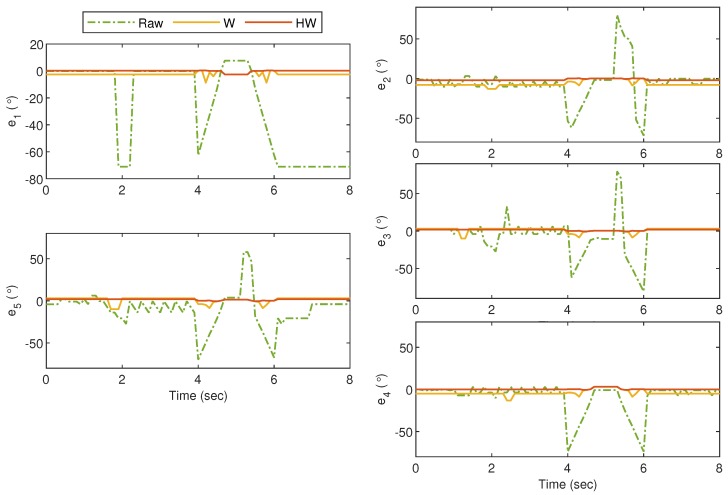
Gesture 1: The corresponding error from the response in Figure 14. The error due to the response from Compensator HW is clearly much lower at all time instances as compared to the others.

For the first experiment, i.e., Gesture 1, the hand gesture starts from the grab position, and all fingers slowly stretch at t≈4 s, remain at this position between t≈4.5 s and t≈5.5 s, and finally return to the grab position at t≥6 s. The values of $\rho_{i}$ for i=1,2,3,4 and 5 are plotted in Figure 14, and it can be clearly observed that without any compensator, the bionic hand fingers are slightly moving when there is no movement from the glove. The movement becomes worse for certain fingers as shown by the large fluctuations of $\rho_{2}$, $\rho_{3}$ and $\rho_{5}$ between t=4 s and t=6 s. Also, $\rho_{1}$ shows an unexpected behavior after t=6 s when the thumb is supposed to bend. These undesired responses can however be alleviated using both Compensators W and HW. It is also clear that the best response is obtained when the goniometric glove is controlled by Compensator HW, particularly during the “grab” instances. To show the error response, we define $e_{i}=\rho_{ri}-\rho_{i}$ which represents the mismatch between the glove’s and the bionic hand’s goniometry. The corresponding $e_{i}$ for Gesture 1 experiment is presented in Figure 15.

**Figure 16:**
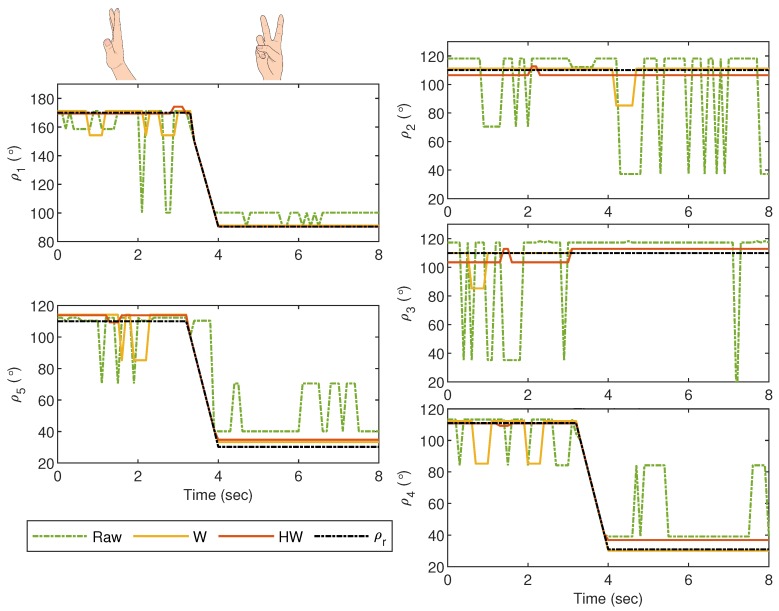
Gesture 2: The mismatch between the glove’s and bionic hand’s goniometry is significantly reduced by using Compensator HW, which also provides a better response as compared to Compensator W, particularly before t=4 s.

**Figure 17:**
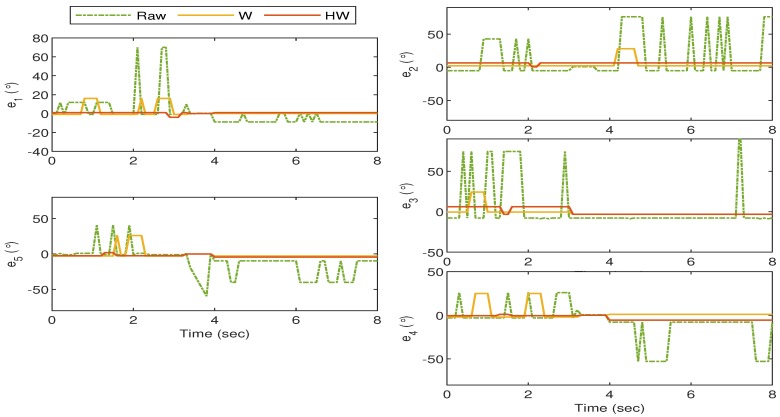
Gesture 2: The corresponding error from the response in Figure 16. The error due to the response from Compensator HW is significantly lower at all time instances as compared to the others.

For Gesture 2 experiment, which is showing the number two sign, the values of $\rho_{i}$ for i=1,2,3,4 and 5 are plotted in Figure 16. The hand gesture starts when all fingers are vertically stretched, and the thumb, middle and pinky fingers slowly flex between t≈2.6 s and t≈4 s. In this experiment, $\rho_{r2}$ and $\rho_{r3}$ are not supposed to vary too much, but large fluctuations in $\rho_{2}$ and $\rho_{3}$ can be seen when there is no compensator applied. A quite similar behavior is also observed after t=4 s for $\rho_{4}$ and $\rho_{5}$, resulting in a large error. In this case, Compensator HW provides a significant improvement as compared to Compensator W due to the erratic readings as seen in $\rho_{1}$, $\rho_{4}$ and $\rho_{5}$ before t=4 s. The error can also be clearly seen from the plot of the corresponding $e_{i}$ in Figure 17.

**Figure 18:**
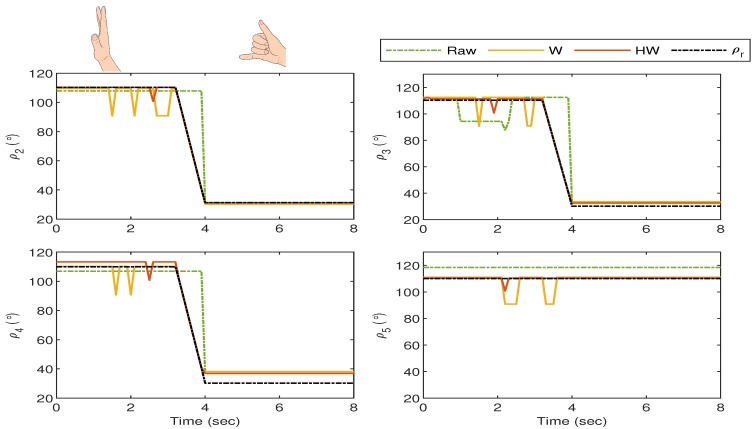
Gesture 3: The mismatch between the glove’s and bionic hand’s goniometry is minimal when Compensator HW is applied as compared to the others.

**Figure 19:**
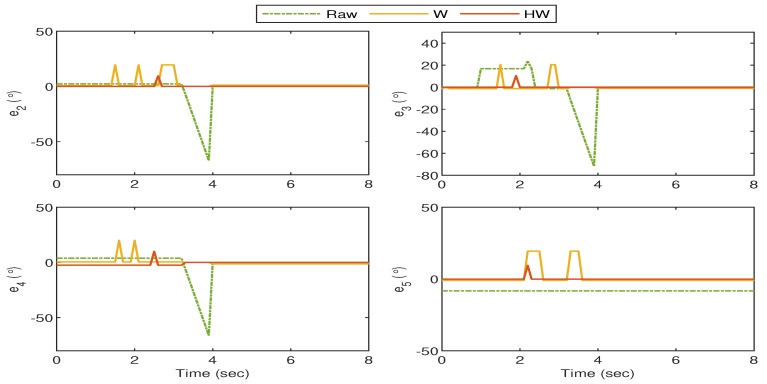
Gesture 3: The corresponding error from the response in Figure 18. The error due to the response from Compensator HW is the least at all time instances as compared to the others.

The bionic hand response for Gesture 3 experiment is shown in Figure 18 where the gesture starts when all fingers are vertically stretched. The pointer, middle and ring fingers start to flex at t≈3.5 s, and the hand stays in the “call sign” gesture after t≈4 s. The figure excludes $\rho_{1}$ as the thumb stays stationary in this gesture, and all responses from “Raw”, “W” and “HW” are very close to $\rho_{r1}$. Similar to the behavior seen from Gesture 2 experiment, Compensator HW outperforms the rest as the fluctuations and the error are minimized as can be observed from $\rho_{i}$ (i=2,3,4 and 5), as shown in Figure 19.

**Figure 20:**
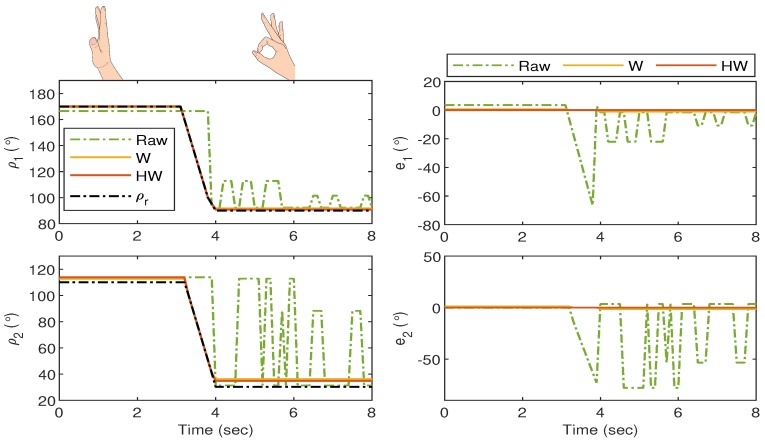
Gesture 4: The mismatch between the glove’s and bionic hand’s goniometry is significantly reduced when both Compensators W and HW are applied.

For Gesture 4, the response is shown in Figure 20 where the gesture starts when all fingers are vertically stretched. The thumb and pointer fingers start to flex at t≈3.5 s, and the hand completely forms the “okay sign” after t≈4 s. In this gesture, the middle, ring, and pinky fingers almost stay stationary and $\rho_{3}$, $\rho_{4}$, $\rho_{5}$ for “Raw”, “W” and “HW” do not show significant deviations from $\rho_{r}$. Hence, only $\rho_{1}$ and $\rho_{2}$ are highlighted in the left column of the figure, together with the corresponding error in the right column. The response shows very large fluctuations when the goniometric glove is not compensated, and the undesired behavior is significantly suppressed by using Compensators W and HW.

The experiments with Gesture 5 and Gesture 6 are slightly different than the previous four gestures as they are designed to analyze the robustness of the proposed strategy when a rapid hand movement is involved. For the Gesture 5 experiment, the hand starts when all fingers are vertically stretched, and then one thumb bends towards the palm, followed by the rest after approximately 0.8 s. The transition proceeds with the pointer until pinky fingers stretch back, close, and stretch again within approximately 2 s. The experiment ends when the thumb is released to its initial position.

**Figure 21:**
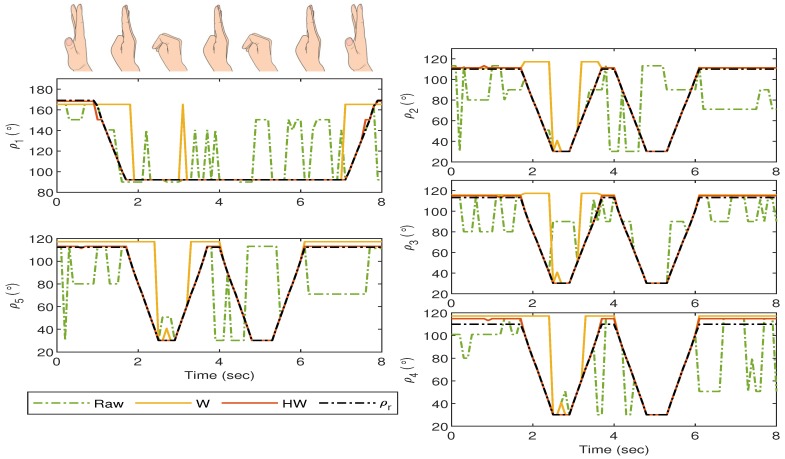
Gesture 5: The mismatch between the glove’s and bionic hand’s goniometry is minimal when Compensator HW is applied as compared to the others.

**Figure 22:**
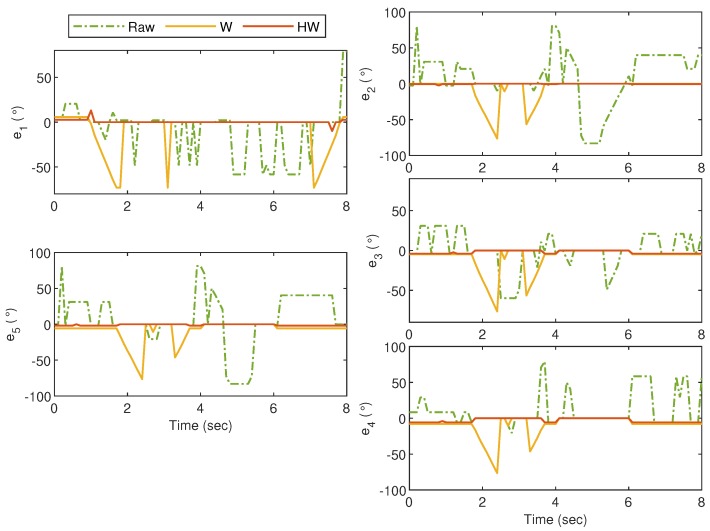
Gesture 5: The corresponding error from the response in Figure 21. The error due to the response from Compensator HW is the least at all time instances as compared to the others.

As can be observed from Figure 21, by using Compensator HW, the mismatch between the glove’s and bionic hand’s goniometry is drastically minimized as compared to that when the raw sensor data or Compensator W are implemented. The is also clearly seen in Figure 22 where the resulting error from Compensator HW implementation does not deviate too much from the zero value.

For the last gesture, which is Gesture 6, the hand starts when all fingers close, but the pointer is stretched away from, and the middle is slightly bent towards the palm. Then the pointer and the middle fingers exchange their positions, followed by all fingers close. The pointer until pinky fingers stretch back and close again within 1 second, and the transition ends when all fingers are released.

**Figure 23:**
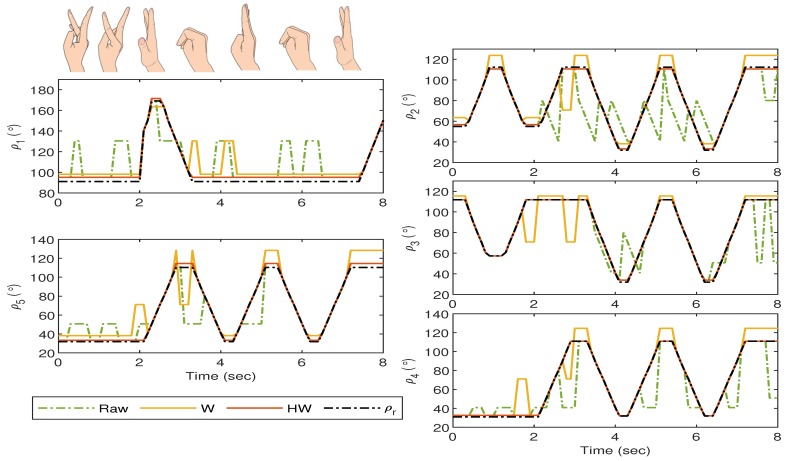
Gesture 6: The mismatch between the glove’s and bionic hand’s goniometry is minimal when Compensator HW is applied as compared to the others.

**Figure 24:**
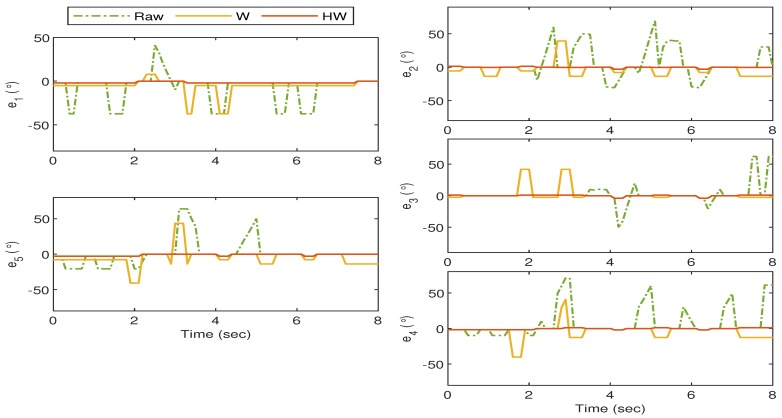
Gesture 6: The corresponding error from the response in Figure 23. The error due to the response from Compensator HW is the least at all time instances as compared to the others.

The responses are recorded in Figure 23, and a similar outcome can still be seen from this last experiment where the implementation of Compensator HW provides the least mismatch between the goniometric glove and the bionic hand. The corresponding error response is shown in Figure 24.

**Figure 25:**
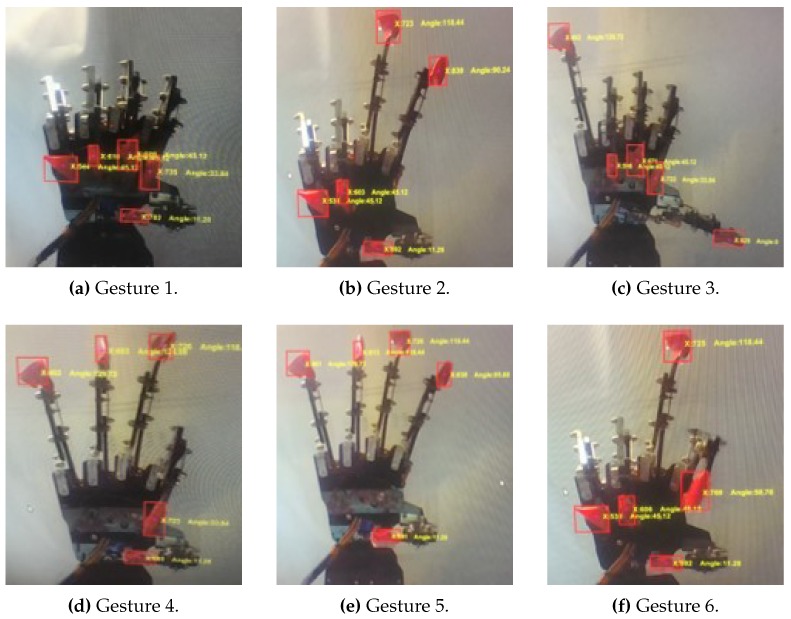
Bionic hand gestures during the image processing in MATLAB from the six experiments.

Some images taken from the camera during the performance evaluations are shown in Figure 25. Each of them illustrates the final position of each finger for each gesture (i.e., grab, number two, call, and okay signs).

As the closed-loop bionic hand system is susceptible to unknown disturbances, the experiments for Gesture 1 until Gesture 6 are repeated for three times to provide a better evaluation, and the performance is quantified in terms of the integral of absolute error, *E* ( ${}^{\circ }$s), as follows:

(9) $$E_{i}=\int_{0}^{t_{f}}\left|e_{i}\left(t\right)\right|dt,\;e_{i}\left(t\right)=\rho_{ri}\left(t\right)-\rho_{i}\left(t\right)$$

where $t_{f}$ denotes the final time of execution. The total error from each finger, which is calculated as

(10) $$E_{T}=\sum_{i=1}^{5}E_{i},$$

for all trials and gestures are recorded in Table 1 when there is no compensation at all, and Table 2 when Compensators W and HW are applied. Table 1 shows average errors between 515${}^{\circ }$s and 1347${}^{\circ }$s for all gestures, which are much bigger than those from Table 2. Interestingly, Compensator HW shows average errors of less than 102${}^{\circ }$s, while the average errors when Compensator W is applied vary between 186${}^{\circ }$s and 370${}^{\circ }$s. This signifies that Compensator HW can provide a major improvement over Compensator W in terms of the temporal mismatch between the goniometric glove’s and the constrained bionic hand’s movements.

## 5. Discussions and Conclusions

This paper has introduced a new control-centric approach to model the characteristic of flex sensors on a goniometric glove, which is designed to capture the user hand gesture that can be used to wirelessly control a bionic hand subject to some constraints. The main technique employs the inverse dynamic model strategy along with a black-box identification for the compensator design, which is aimed to provide an approximate linear mapping between the raw sensor output and the dynamic finger goniometry. To smoothly recover the goniometry on the bionic hand’s side during the wireless transmission, the compensator is restructured into a Hammerstein–Wiener model, which consists of a linear dynamic system and two static nonlinearities. The linear system is constructed by simplifying the dynamic model from the inverse dynamic design technique, while the static nonlinearities are introduced based on the constraints of the bionic hand, and to account for the uncertain behavior of the sensors as well as the unmodeled dynamics from the black-box identification method. A series of real-time experiments involving several hand gestures have been conducted to analyze the performance of the proposed method. The experimental results with several trials for each gesture show that a great improvement is obtained via the Hammerstein–Wiener compensator approach where the resulting average errors are significantly smaller than the other two methods considered. This concludes that the proposed strategy can remarkably improve the dynamic goniometry of the glove, and thus, provides a smooth human–robot collaboration with the bionic hand.

While the experimental results show a great accuracy via the proposed method, this work only considers one degree-of-freedom movement from the MCP joint. For future work, the framework will be extended to include the overall 3D motion of the goniometric glove to further enhance the bionic hand control system. This however may require some modifications on the bionic hand’s structure to allow more gestures from the glove to be recovered. The proposed method can also be combined with another technique such as artificial neural network to find the correlations between the hand palm and the fingers, as well as the correlation between the fingers itself.

## Figures and Tables

**Table 1:** Total error, $E_{T}$ (${}^{\circ }$s), for each gesture and its average value when there is no compensator on the goniometric glove.

Gesture	1	2	3	4	5	6
Trial 1	1036.2	1701.5	2014.2	1201.2	1479	418.5
Trial 2	545.71	654.14	1023.1	721.78	1080.25	525.3
Trial 3	461.21	512.23	1001.2	657.12	700.23	602.3
Average	681.04	956.0	1346.2	860.0	1086.5	515.4

**Table 2:** Total error, $E_{T}$ (${}^{\circ }$s), for each gesture and its average value when Wiener and Hammerstein–Wiener compensators are applied on the goniometric glove.

	Wiener						Hammerstein–Wiener					
Gesture	1	2	3	4	5	6	1	2	3	4	5	6
Trial 1	354.2	412.21	401.28	70.254	299.5	315.9	136.7	97.75	254.3	76.76	39.47	48.4
Trial 2	144.25	152.25	101.25	98.321	441.2	401	49.8	39.1	39.34	37.03	108.3	81.3
Trial 3	60.214	101.27	124.27	452.12	300.2	389.3	6.131	6.137	12.08	3.283	85.3	104.9
Average	186.25	221.91	208.93	206.90	347	368.7	64.21	47.66	101.90	39.02	77.69	78.2

## Notations and Abbreviations

The following notations and abbreviations are used in this manuscript:

*i*	The subscript i=1,2,3,4 and 5 on a symbol indicates the signal associated with the thumb, pointer, middle, ring, and pinky fingers, respectively.
$\beta_{i}$	input signal to the bionic hand’s system
MCP	metacarpophalangeal
DIP	distal interphalangeal
PIP	proximal interphalangeal
$\theta_{i}^{D}$	angle measured at the DIP joint of the bionic hand
$\theta_{i}^{P}$	angle measured at the PIP joint of the bionic hand
$\theta_{i}$	angle measured at the MCP joint of the bionic hand (without constraint)
$\theta_{L}$, $\theta_{U}$	lower and upper bounds of $\theta_{i}$
$\psi_{i}$	the constraint imposed on $\theta_{i}$
$\rho_{i}$	angle measured at the MCP joint of the bionic hand (with constraint)
$\rho_{ri}$	angle measured at the MCP joint of the goniometric glove
$e_{i}$	error or mismatch between $\rho_{i}$ and $\rho_{ri}$
$\alpha_{i}$	raw sensor value
$\phi_{wi}$	static nonlinearity after the compensator’s dynamic model
$\phi_{hi}$	static nonlinearity before the compensator’s dynamic model
$\sigma_{wi}$	output of the compensator’s dynamic model
$\sigma_{hi}$	input of the Hammerstein–Wiener compensator’s dynamic model
$d_{in}$	unknown input disturbance within the bionic hand system
*P*	dynamic model of the Wiener compensator
$P_{hw}$	dynamic model of the Hammerstein–Wiener compensator
μC1	microcontroller for the goniometric glove
μC2	microcontroller for the bionic hand
$E_{i}$	integral of absolute error
$t_{f}$	final time of execution
$E_{T}$	total error from each finger
